# Circulating Fetuin-A and Risk of All-Cause Mortality in Patients With Chronic Kidney Disease: A Systematic Review and Meta-Analysis

**DOI:** 10.3389/fphys.2019.00966

**Published:** 2019-07-30

**Authors:** Zhongwei Zhou, Yuqiao Ji, Huixiang Ju, Hongmei Chen, Mingzhong Sun

**Affiliations:** Department of Clinical Laboratory, School of Medicine, Affiliated Yancheng Hospital, Southeast University, Yancheng, China

**Keywords:** fetuin-A, chronic kidney disease, mortality, dialysis, meta-analysis

## Abstract

**Background:** Investigations on the association of circulating fetuin-A with all-cause mortality risk in patients with chronic kidney disease (CKD) are conflicting. This meta-analysis aimed to provide a comprehensive estimation of the relationship between fetuin-A and all-cause mortality in CKD patients.

**Methods:** A systematic literature search was performed in PubMed, EMBASE, and The Cochrane Library up until 12 December 2018. Hazard risk (HR) and 95% confidence interval (CI) were pooled using random-effect or fixed-effect model models.

**Results:** A total of 13 studies comprising 5,169 CKD patients were included in the meta-analysis. In a comparison of individuals in the bottom third vs. the top third of baseline fetuin-A levels, the pooled multivariate-adjusted HR for the risk of all-cause mortality was 1.92 (95% CI 1.31–2.80), and the significant association was observed only in dialysis patients, but not non-dialysis patients. When fetuin-A was treated as continuous variables, per 0.1 g/L increase of fetuin-A levels was associated with a 8% lower mortality risk in dialysis patients (HR 0.92, 95% CI 0.87–0.97, *p* = 0.001), but per 0.01 g/L was not. Sensitivity analysis indicated the association was not adjusted by diabetes and inflammation.

**Conclusion:** Lower fetuin-A levels are associated with an increased risk of all-cause mortality independent of diabetes and inflammation in dialysis patients, and there may be a dose-response relationship between them.

## Introduction

Chronic kidney disease (CKD) is a major public health concern worldwide, affecting 11 to 13% of global population, and it is associated with adverse clinical events such as cardiovascular disease (CVD) and progression to end-stage renal disease (ESRD) (Hill et al., 2016). There is growing awareness that individuals with CKD are at increased risk of all-cause and cardiovascular mortality, especially for patients who have progressed to ESRD (Bochud, 2015; Romagnani et al., 2017). Although traditional markers such as estimated glomerular filtration rate (eGFR) and albuminuria play effective roles in prognostic evaluation of CKD patients (Liu et al., 2014; Wang et al., 2017), novel prognostic biomarkers have been explored which may improve current risk stratification system and provide new insights into the mechanisms of the development and progression of CKD (Mihai et al., 2016; Homsak and Ekart, 2018).

Fetuin-A, which is a multifunctional glycoprotein produced by liver cells and then secreted into the blood circulation, is a potent inhibitor of vascular calcification (Herrmann et al., 2012). There is a high prevalence of vascular calcification which is associated with increased mortality in CKD patients (Sigrist et al., 2007; Jean et al., 2015; Vervloet and Cozzolino, 2017). Therefore, there are plausible biological mechanisms by which fetuin-A may be implicated in the associated risks of mortality in individuals with CKD. However, studies published to date yielded inconsistent findings, with variables adjustment for potential confounders (Stenvinkel et al., 2005; Ix et al., 2007; Jung et al., 2011; Alderson et al., 2016), and so there remains uncertainty whether circulating fetuin-A levels are independently associated with risks of mortality in CKD patients.

To address this issue, we conducted a systematic review and meta-analysis to summarize and synthesize published data on the relationship of circulating fetuin-A levels with all-cause mortality in CKD patients with or without undergoing dialysis.

## Materials and Methods

The protocol of this meta-analysis was conducted in accordance with the guidelines of the Meta-analysis of Observational Studies in Epidemiology (MOOSE) group (Stroup et al., 2000) and the preferred reporting items for systematic reviews and meta-analyses (Moher et al., 2009). Since this study is based on the literature review, it is not involved in ethics issue.

### Search Strategy

We systematically searched PubMed, EMBASE, and The Cochrane Library for prospective observational studies up to 12 December 2018. The search terms included: (fetuin-A OR alpha-2-HS-Glycoprotein OR AHSG) AND (“chronic kidney disease” OR “end-stage renal disease” OR hemodialysis OR “peritoneal dialysis” OR dialysis) AND (mortality OR death). In addition, the references from these relevant articles were manually searched for additional eligible studies.

### Study Selection

Studies were selected if they met the following criteria: (1) prospective observational studies; (2) study populations should be CKD patients with or without undergoing dialysis; (3) baseline circulating fetuin-A levels as exposure; (4) outcome measures included all-cause mortality; (5) the multivariate-adjusted hazard ratio (HR) or risk ratio (RR) with [95% confidence interval (CI)] was provided. Studies only reporting unadjusted risk estimates were excluded.

### Data Extraction and Quality Assessment

Two investigators independently selected studies and extracted the following information: first author's name, publication year, study origin, type of patients, sample size, mean age or age range, percentage of male patients, measurements of risks, number of death event, the most fully adjusted risk estimate, follow-up duration, and adjustment for potential cofounders in multivariate model. Any disagreement in opinion in the data extraction was resolved by discussion among all researchers. For the methodological quality evaluation, Newcastle-Ottawa Scale (NOS) for cohort studies was used for these included studies (Stang, 2010). The NOS was based on the following three aspects: selection, comparability and outcome, and a maximum rating of 9 stars may be given to individual studies according to this scale. A study with a score ≥8 stars would be considered as a high-quality study, and 6–7 stars as a moderate quality study.

### Statistical Analysis

All statistical analyses were performed using the Stata15.0 software package (Stata Corp LP, College Station, TX, USA). The included studies reported RR, which were assumed to approximate HR and were combined directly. Individual studies reported the multivariate-adjusted risk estimate in this meta-analysis were displayed in different variable types [for example, comparing lower vs. upper half, tertile, quartile, or per one standard deviation (SD), per 0.01 unit and per 0.1 unit change]. In order to provide a consistent comparison to meta-analysis, we used previously described methods to transform some of these types (comparing lower vs. upper half, quartile and per one SD change) to a measure of association corresponding to the bottom vs. the top third of the baseline fetuin-A concentration (Danesh et al., 1998). Briefly, we transformed log HR by assuming a normal distribution, and the uniform scale can be estimated as a scaling factor of 2.18 divided by 1.59 times the log HR for comparison of the lower vs. upper half, and 2.18 divided by 2.54 times for comparison of the bottom and top quartiles. For studies where associations were reported one SD change, 2.18 was used as the scaling factor for the log HR. While some studies in which associations were reported per 0.01 unit and per 0.1 unit change were inappropriate to transform based on the above methods, they were pooled separately. Pooled effect sizes were presented as the HR with 95% CI. Statistical heterogeneity among studies was assessed using the *x*^2^ test with significance set at <0.10, and quantified by the *I*^2^ index (Higgins et al., 2003). A value of *I*^2^ > 50% would indicate substantial heterogeneity. When significant heterogeneity was observed, studies were pooled using a random effect model; otherwise, a fixed-effect model was selected. This is because the random-effect model is a more conservative approach which yields a wider CI than fixed effect model if there is a significant heterogeneity between studies (Masi et al., 2015). To explore the potential source of heterogeneity and obtain further information from different subpopulations, subgroup analyses were performed according to the study region (non-European or European), type of patients (dialysis or non-dialysis), sample size (<200 or ≥200) and duration of follow-up (<3 years or ≥3 years). Sensitivity analysis was performed to evaluate the impact of anyone study on the overall results by omitting one study at each turn. Potential publication bias was explored by visual inspection of the funnel plot and analysis with Egger's test.

## Results

### Literature Search

**Figure 1:**
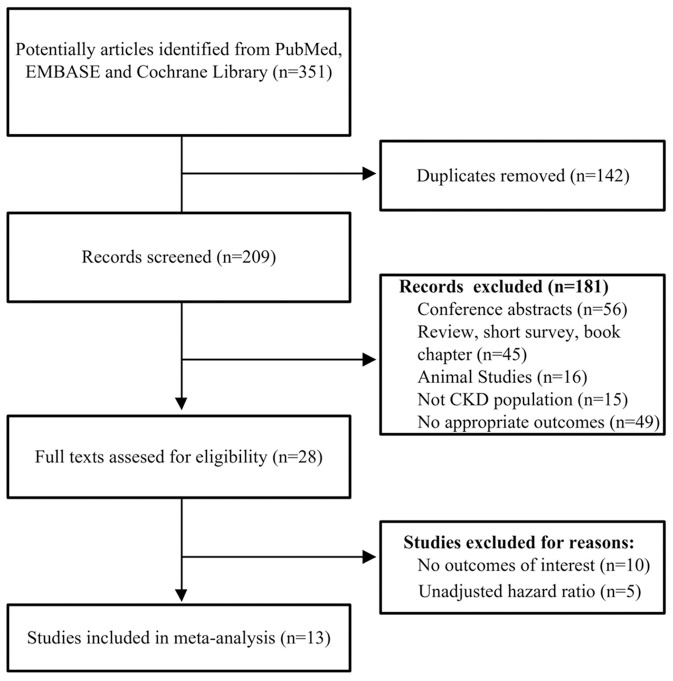
Flow chart of the study selection process.

A flowchart of the included and excluded studies is showed in Figure 1. A total of 351 potentially relevant records were identified after an initial search from the selected electronic databases. We first removed 142 literatures due to duplication, and then removed further 181 records based on the screening of titles and abstracts. The remaining 28 articles were identified for full-text analysis. We further excluded 10 studies for no outcomes of interest, and 5 only reporting unadjusted HR. Finally, 13 studies (Stenvinkel et al., 2005; Wang et al., 2005, 2008; Honda et al., 2006; Hermans et al., 2007; Ix et al., 2007; Carrero et al., 2008; Metry et al., 2008; Jung et al., 2011; Verduijn et al., 2011; Chen et al., 2014; Scialla et al., 2014; Alderson et al., 2016) met the criteria and were selected for the final analysis.

### Characteristics of Included Studies

Table 1 summarized the baseline characteristics of the included studies. The 13 included studies were published from 2005 to 2016 and involved a total of 5,169 patients with CKD. Individual study populations ranged from 58 to 987. Among these, two studies (Ix et al., 2007; Alderson et al., 2016) were limited to non-dialysis CKD patients, and three studies (Carrero et al., 2008; Metry et al., 2008; Chen et al., 2014) enrolled hemodialysis patients and two studies (Wang et al., 2005, 2008) peritoneal dialysis patients, while the other six studies (Stenvinkel et al., 2005; Honda et al., 2006; Hermans et al., 2007; Jung et al., 2011; Verduijn et al., 2011; Scialla et al., 2014) included both hemodialysis and peritoneal dialysis patients. The duration of follow-up ranged from 2.14 to 9.5 years. Seven studies were conducted in European countries including UK (Alderson et al., 2016), Sweden (Stenvinkel et al., 2005; Honda et al., 2006; Carrero et al., 2008; Metry et al., 2008) and the Netherlands (Hermans et al., 2007; Verduijn et al., 2011), four in Asian countries including China (Wang et al., 2005, 2008), Korea (Jung et al., 2011) and Taiwan (Chen et al., 2014), and two in the USA (Ix et al., 2007; Scialla et al., 2014). All the included studies were classified as moderate to high quality according to NOS scale (ranging from 6 to 9 stars).

**Table 1:** Summary of clinical studies included in meta-analysis.

**References**	**Region**	**Type of patients**	**Sample size** **(% male)**	**Age (years)**	**Measurements of risks**	**Number of death/HR** **(95% CI)**	**Follow-up (years)**	**Adjustment for covariates**	**Overall NOS**
Stenvinkel et al., 2005	Sweden	HD and PD	258 (62.4)	52 ± 1	Low vs. high	Total death: 88; HR (95% CI): 2.58 (1.64–4.07)	3.5	Age, sex, diabetes, CVD, smoking, dialysis modality, calcium phosphate product, hypoalbuminemia, CRP	9
Honda et al., 2006	Sweden	HD and PD	176 (64.8)	54 ± 12	Lowest quartile 4 vs. highest	Total death: 42; HR (95% CI): 3.20 (1.00–10.40)	2.14	Age, sex, diabetes	6
Wang et al., 2005	China	PD	238 (51.3)	56 ± 12	Per 0.01 g/L increase	Total death: 89; HR (95% CI): 0.98 (0.94–1.02)	2.64	Age, diabetes, residual GFR and AVD, cardiac valvular calcification, CRP, serum albumin	7
Ix et al., 2007	USA	Non-dialysis CKD	822 (60.0)	52 ± 12	Highest tertile 3 vs. lowest	Total death: 204; HR (95% CI): 1.01 (0.71–1.46)	9.5	Age, sex, ethnicity, GFR, history of diabetes, hypertension, log CRP, proteinuria, randomized blood pressure, protein intake	9
Hermans et al., 2007	The Netherlands	HD and PD	987 (59.0)	60 ± 14	Per 0.1 g/L increase	Total death: 396; HR (95% CI): 0.91 (0.84–0.99);	2.8	Age, sex, diabetes, primary kidney disease, CVD, dialysis modality, smoking, log hsCRP, albumin, subjective global assessment, BMI, calcium, phosphate	8
Metry et al., 2008	Sweden	HD	222 (55.4)	63 ± 14	Low vs. high	Total death: 85; HR (95% CI): 2.3 (1.2–4.5)	2.58	Age, sex, comorbidity risk groups, dialysis vintage and CRP	7
Wang et al., 2008	China	PD	231 (51.1)	56 ± 12	Low vs. high	Total death: 66; HR (95% CI): 3.30 (1.44–7.52)	3.0	Age, sex, diabetes, duration of dialysis, hypertension, background atherosclerotic vascular disease, residual GFR, hemoglobin, albumin, LDL-C, CRP, IL-6	9
Carrero et al., 2008	Sweden	HD	175 (56.0)	66 (23–86)	Low vs. high	Total death: 70; HR (95% CI): 1.57 (0.94–2.58)	2.58	Age, sex, IL-6, leucocyte telomere length	6
Jung et al., 2011	Korea	HD and PD	58 (na)	na	Per SD increase	Total death: 27; HR (95% CI): 0.34 (0.17–0.67)	3.83	Age, sex, diabetes, history of CVD, smoking, BMI, albumin, log CRP	6
Verduijn et al., 2011	The Netherlands	HD and PD	549 (na)	na	Per 0.1 g/L increase	Total death: 213; HR (95% CI): 0.89 (0.80–1.00)	5.0	Age, sex, dialysis modality, CVD, diabetes, BMI, hypercholesterolaemia, hs-CRP	7
Chen et al., 2014	Taiwan	HD	388 (48.0)	59 ± 12	Per 0.01 g/L increase	Total death: 92; HR (95% CI): 0.97 (0.91–0.99);	3.4	Age, sex, HD vintage; diabetes, hypertension, concurrent CV disease, hemoglobin, calcium phosphate product, hs-CRP	9
Scialla et al., 2014	USA	HD and PD	602 (53.2)	57.8 ± 14.9	Highest tertile 3 vs. lowest; Per 0.1 g/L increase	Total death: 423; HR (95% CI) for highest tertile 3 vs. lowest: 0.77 (0.57–1.04); HR (95% CI) for per 0.1 g/L increase: 0.94 (0.87–1.02)	3.4	Age, sex, race, index of coexistent disease, diabetes, CVD, BMI, phosphate, calcium, serum albumin, log IL-6, log CRP, log FGF-23	9
Alderson et al., 2016	UK	Non-dialysis CKD	463 (61.8)	63.8 ± 14.1	Per SD increase	Total death: 217; HR (95% CI): 1.01 (0.99–1.01)	3.83	Age, sex, history of CVD, diabetes, smoking, SBP, calcium, phosphorus, albumin, hemoglobin, Hemoglobin, FGF-23, osteoprotegerin	8

*HR, hazard ratio; CI, confidence interval; HD, hemodialysis; PD, peritoneal dialysis; CVD, cardiovascular disease; CRP, C-reactive protein; GFR, glomerular filtration rate; AVD, atherosclerotic vascular disease; CKD, chronic kidney disease; hsCRP, high sensitivity C-reactive protein; BMI, body mass index; LDL-C, low density lipoprotein cholesterin; IL-6, interleukin-6; SBP, systolic blood pressure; NOS, Newcastle Ottawa Scale*.

### The Association of Fetuin-A and All-Cause Mortality

**Figure 2:**
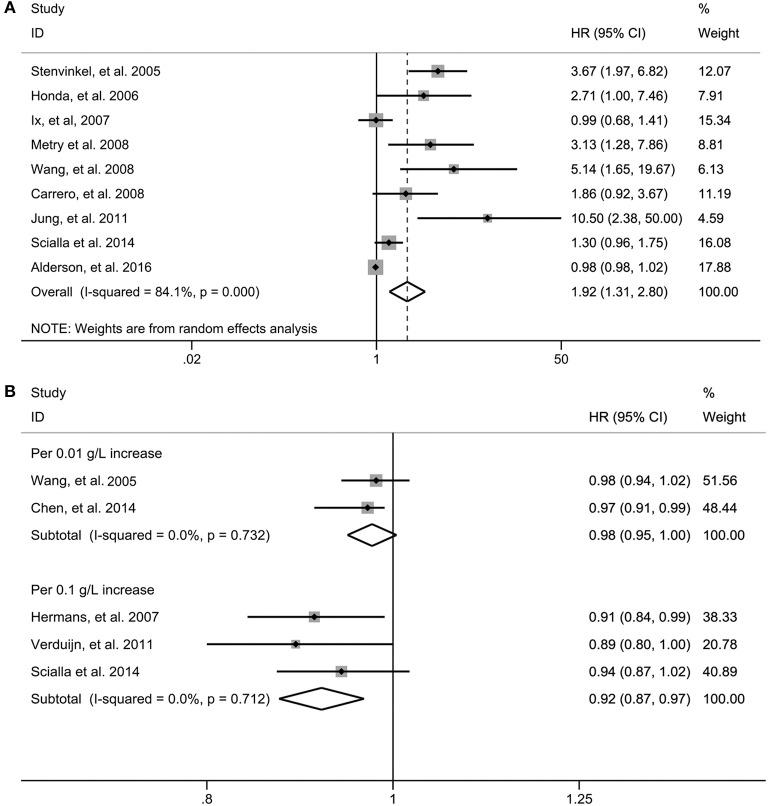
Risk estimate for all-cause mortality in individuals in the bottom compared with the top third of fetuin-A levels in eligible studies **(A)**. Risk estimate for all-cause mortality in fetuin-A levels for per 0.1 g/L and per 0.01 g/L increase in eligible studies **(B)**. HR, hazard risk; CI, confidence interval.

In the pooled analysis of nine studies (Stenvinkel et al., 2005; Honda et al., 2006; Ix et al., 2007; Carrero et al., 2008; Metry et al., 2008; Wang et al., 2008; Jung et al., 2011; Scialla et al., 2014; Alderson et al., 2016) where the association between fetuin-A and risk of all-cause mortality was presented as the bottom vs. the top third, the pooled HR was 1.92 (95% CI 1.31–2.80, *p* = 0.001). A random effect model was selected because there was significant heterogeneity among the included studies (*I*^2^ = 84.1%, *p* < 0.001) (Figure 2A).

When three studies (Hermans et al., 2007; Verduijn et al., 2011; Scialla et al., 2014) in which the association were presented as per 0.1 g/L increase in circulating fetuin-A levels were pooled, we found that the risk of all-cause mortality decreased by 8% (HR 0.92, 95% CI 0.87–0.97, *p* = 0.001); however, per 0.01 g/L increase of fetuin-A levels [two studies (Wang et al., 2005; Chen et al., 2014)] were not significantly associated with decreased all-cause mortality (HR 0.98, 95% CI 0.95–1.00; *p* = 0.092) (Figure 2B). Fixed-effect models were used for the two measurements with no evidence of significant heterogeneity.

### Subgroup Analysis

**Figure 3:**
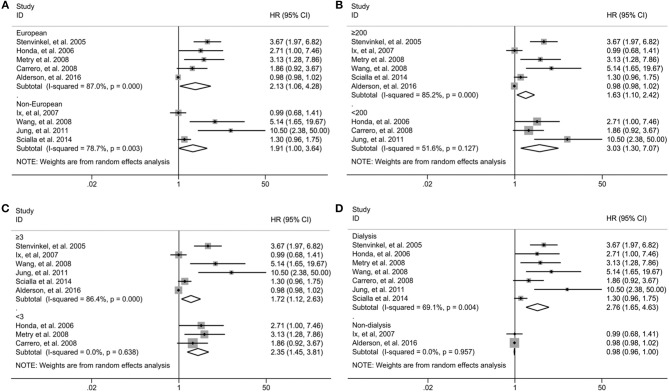
Subgroup analysis for all-cause mortality in individuals in the bottom compared with the top third of fetuin-A levels when stratified by the study region **(A)**, sample sizes **(B)**, type of patients **(C)**, and duration of follow-up **(D)**. HR, hazard risk; CI, confidence interval.

Subgroup analysis was performed on all-cause mortality by the study region, sample sizes, duration of follow-up and type of patients in nine studies in which the association was presented as the bottom vs. the top third. As shown in Figure 3, the association between fetuin-A levels and the risk of all-cause mortality was not modified by study region (Figure 3A), sample sizes (Figure 3B), and duration of follow-up (Figure 3C) except different type of patients (Figure 3D). Significant association between low fetuin-A levels and higher risk of mortality was observed in dialysis patients (HR 2.76, 95% CI 1.65–4.63), but not in non-dialysis patients (HR 0.98, 95% CI 0.96–1.00). Although no heterogeneity was observed in individuals with <3 years of follow-up (*I*^2^ = 0) and non-dialysis patients (*I*^2^ = 0) stratified by the follow-up period and type of patients, considerable heterogeneity was still found in other subgroups (all *I*^2^ > 50%).

### Publication Bias and Sensitivity Analysis

**Figure 4:**
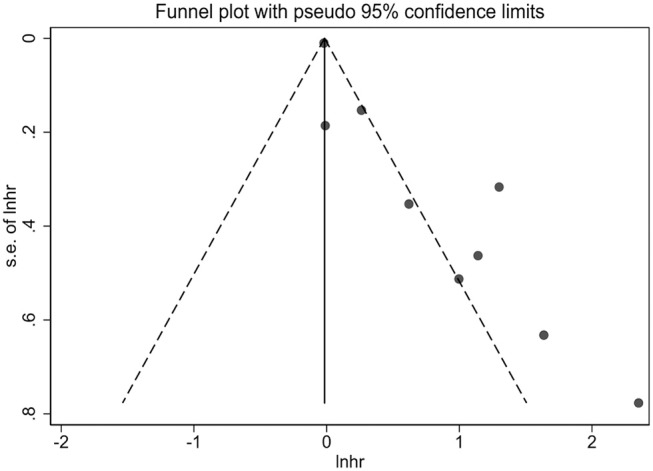
Funnel plot for potential publication bias between fetuin-A levels (the bottom vs. the top third) and all-cause mortality in eligible studies.

Due to the fact that the small number of studies were included in measurements of per 0.1 g/L and per 0.01 g/L increase of fetuin-A levels, publication bias and sensitivity analysis were performed only in 9 studies in which the association was presented as extreme thirds. Publication bias was assessed first by a visual inspection of funnel plot, which showed asymmetrical (Figure 4). Egger's test further showed that there may be potential publication bias (*p* = 0.001).

**Figure 5:**
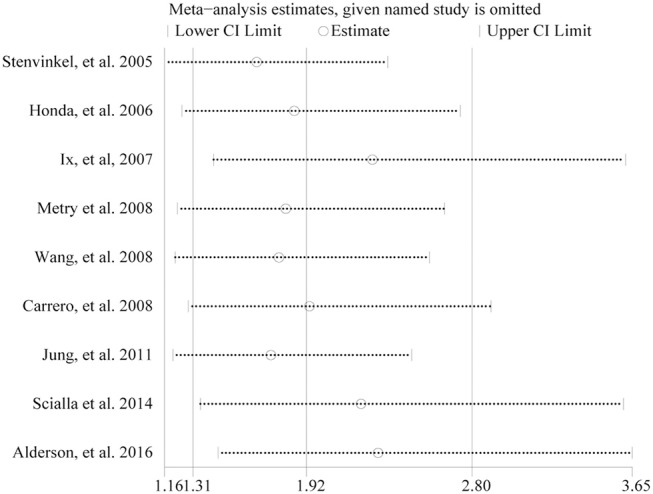
Sensitivity analysis between fetuin-A levels (the bottom vs. the top third) and all-cause mortality in eligible studies.

In the sensitivity analysis, pooled HR varied within 1.65–2.30, and low 95% CI varied within 1.16–1.45 when anyone study was omitted in 9 studies (Figure 5). In addition, the association between low fetuin-A levels and higher risk of mortality remained significant after excluding two studies (Carrero et al., 2008; Metry et al., 2008) which did not adjust for diabetes (HR 3.15, 95% CI 1.50–6.62), or one study (Honda et al., 2006) which did not adjust for inflammatory cytokines (HR 2.81, 95% CI 1.57–5.04) in dialysis patients.

## Discussion

This is the first meta-analysis reporting pooled data on the association between circulating fetuin-A levels and all-cause mortality in CKD patients. The data suggest that CKD patients with fetuin-A levels in the bottom third had a 92% greater risk of all-cause mortality compared with those in the top third. In subgroup analysis, the significant association between low fetuin-A levels and higher risk of mortality was observed only in dialysis patients, but not non-dialysis patients. Furthermore, we found that per 0.1 g/L increase of fetuin-A levels was associated with a 8% lower risk of all-cause mortality in dialysis patients, but per 0.01 g/L was not. These findings suggest that lower levels of fetuin-A were a significant predictor of all-cause death in dialysis patients, and there may be a dose-response relationship between them.

In this meta-analysis, in order to determine the independent association between fetuin-A and all-cause mortality, we only included studies that reported the multivariate-adjusted risk estimates, but not unadjusted ones. Circulating fetuin-A levels can be influenced by many factors such as insulin resistance, glucolipid metabolism and systemic inflammation. There are interactions between fetuin-A and free fatty acid which induces inflammatory signaling and insulin resistance, and subsequently leads to increased risk of type 2 diabetes (T2DM) (Pal et al., 2012; Stefan and Haring, 2013). Several meta-analyses have indicated higher circulating fetuin-A levels were associated with increased risk of T2DM (Guo et al., 2018; Roshanzamir et al., 2018; Sujana et al., 2018). But it seems that the association between fetuin-A and inflammation is more complex. The expression of fetuin-A may be diversely regulated by different pro-inflammatory mediators, which was suppressed by TNF and IFN-γ, but promoted by high mobility group box-1 (HMGB-1), and therefore it functions as a positive or negative acute phase proteins (APP) in injury and infection (Wang and Sama, 2012). During some inflammatory diseases such as CKD, fetuin-A was viewed more as a negative APP (de Jager et al., 2014). Several studies included in this meta-analysis showed that there were significantly negative correlations between fetuin-A and high sensitivity C-reactive protein (hsCRP) and interleukin-6 (Wang et al., 2008; Verduijn et al., 2011), and in particular, one study indicated that low levels of fetuin-A were associated with increased mortality in dialysis patients only in the presence of inflammation (Metry et al., 2008). Therefore, to confirm whether diabetes and inflammation as confounding factors may influence the association between fetuin-A and the risk of all-cause mortality, we excluded two studies (Carrero et al., 2008; Metry et al., 2008) which did not adjust for diabetes, or one study (Honda et al., 2006) which did not adjust for inflammatory cytokines in the sensitivity analyses, and found the association between them remained significant. These findings suggest that lower fetuin-A levels are associated with an increased risk of all-cause mortality independent of diabetes and inflammation in dialysis patients.

The association between lower fetuin-A levels and poor prognosis in CKD patients could be explained by several possible mechanisms. First, fetuin-A can inhibit the deposition of calcium phosphate by forming soluble mineral complexes, which can be called as fetuin-mineral complex (Herrmann et al., 2012); therefore, decreased fetuin-A levels could lead to a decline in inhibitory effect on vascular calcification which is a progressive complication and a marker of poor prognosis in CKD patients. Animal studies have shown that fetuin-A deficiency in DBA/2 mice spontaneously develop widespread soft tissue calcification, including significant renal calcification (Schafer et al., 2003); and in mouse models with CKD, fetuin-A deficiency and hyperphosphatemia have also been shown to have synergistic effects in the pathogenesis of extraosseous calcification (Westenfeld et al., 2007). Second, *in vitro* studies suggest that fetuin-A can enhance phagocytosis of apoptotic cells and macropinocytosis by human macrophages (Jersmann et al., 2003); thus, low fetuin-A levels may weaken the phagocytic function, which led to infection or inflammation. Persistent inflammation not only itself acts as a risk factor, but also it is seen as a promoter in development of CKD (Carrero and Stenvinkel, 2009; Miyamoto et al., 2011). Recently, targeting inflammation has been used as a new therapeutic means in CKD patients (Impellizzeri et al., 2014). Finally, fetuin-A can be considered as a biomarker of nutritional status (Perez-Sotelo et al., 2017), and malnutrition is a common complication and associated with increased mortality in CKD patients (Ignjatovic et al., 2013). Dialysis patients have usually much poorer nutritional status than non-dialysis patients (Dai et al., 2017), which may be one of the reasons that low fetuin-A levels can predict the mortality only in dialysis patients in this meta-analysis. One study (Honda et al., 2006) included in this meta-analysis also showed that there is an interaction between fetuin-A and nutritional status which alters cardiovascular outcomes and survival in hemodialysis patients.

Several limitations of this meta-analysis should be of concern. First, the accumulating evidence showed that individuals with CKD are at higher risk of cardiovascular mortality, and several studies included this meta-analysis also analyzed the relationship of circulating fetuin-A levels with cardiovascular mortality (Stenvinkel et al., 2005; Hermans et al., 2007; Ix et al., 2007; Chen et al., 2014; Scialla et al., 2014). However, this meta-analysis only investigated the association between fetuin-A and all-cause mortality, mainly because the number of studies that concentrated on the relationship between fetuin-A and cardiovascular mortality was relatively limited, especially on their relationship after multivariate adjustment for confounders. The limited number of studies was also evidenced in stratified subgroups and the measurements of per 0.1 and per 0.01 g/L. Therefore, further large-scale studies are required to explore the relationship between fetuin-A and cardiovascular mortality, fetuin-A and all-cause mortality in non-dialysis patients and the dose-response relationship between them. Second, there was statistical heterogeneity in the pooled outcome of extreme thirds. Although subgroup analyses were performed, and type of patients and follow-up duration were shown to be associated with heterogeneity at some level, the high levels of heterogeneity in most subgroups still cannot be reasonably explained. In addition, as limited information available from the included studies, it is not sure whether some other factors could contribute to the between-study heterogeneity. Third, some other potential confounders such as dialysis vintage are limited in the eligible studies included in this meta-analysis, which prevented us from further analyzing whether these confounding factors had moderating effects on the pooled results. Moreover, although we only included studies that reported the multivariate-adjusted risk estimates, the adjusted confounders were different across studies included in this meta-analysis. For example, one study (Honda et al., 2006) adjusted only for age, sex and diabetes. Therefore, the limited information and inadequate adjustment for potential confounding factors may overestimate the risk estimate. Finally, the asymmetric funnel plot suggested that the potential publication bias may be due to small study effects. In this meta-analysis, we did not include conference abstracts which might lead to missing some small and unpublished studies. However, it should be recognized that the asymmetric funnel plot is not necessarily originated from publication bias but can also be created by significant heterogeneity among studies (Terrin et al., 2003). In addition, sensitivity analysis indicated that no individual study significantly influenced the association between low fetuin-A levels and higher risk of mortality.

In conclusion, this meta-analysis demonstrated that lower fetuin-A levels are associated with an increased risk of all-cause mortality independent of diabetes and inflammation in dialysis patients, and there may be a dose-response relationship between them. Further large-scale studies are required to confirm these findings.

## Data Availability

All datasets generated for this study are included in the manuscript and/or the supplementary files.

## Author Contributions

ZZ contributed to statistical analyses and paper writing. YJ and HC contributed to literature search, data extraction, and quality assessment. HJ contributed to editing and revision of the manuscript. MS designed the study and performed data interpretation. All authors have approved the final version of this paper that was uploaded to the journal website.

### Conflict of Interest Statement

The authors declare that the research was conducted in the absence of any commercial or financial relationships that could be construed as a potential conflict of interest.
